# Microstructural Integrity of the Hippocampus During Childhood: Relations With Age and Source Memory

**DOI:** 10.3389/fpsyg.2020.568953

**Published:** 2020-09-16

**Authors:** Daniel D. Callow, Kelsey L. Canada, Tracy Riggins

**Affiliations:** ^1^Department of Kinesiology, University of Maryland, College Park, MD, United States; ^2^Program in Neuroscience and Cognitive Science, University of Maryland, College Park, MD, United States; ^3^Department of Psychology, University of Maryland, College Park, MD, United States

**Keywords:** hippocampus, microstructure, diffusion MRI (DWI), source memory task, early childhood

## Abstract

The hippocampus is a brain structure known to be important for memory. However, studies examining relations between hippocampal volume and memory across development yield mixed results. This may be due in part to the fact that volume is a coarser measure of hippocampal composition. Studies have begun to examine measures of diffusion, which capture characteristics of the microstructure of the hippocampus, and thus may provide additional information about the integrity of the underlying neural circuits. The present study applied this approach to a developmental period characterized by dramatic changes in both hippocampal microstructure and memory behavior – early childhood. Specifically, measures of hippocampal microstructural integrity were related to age and source memory performance in 93 children aged 4–8 years. Results revealed significant negative associations between hippocampal mean diffusivity and both age and memory, even after controlling for differences in hippocampal volume. These results suggest that hippocampal diffusion may provide additional, independent information about hippocampal integrity compared to volume, particularly during early childhood when important developmental changes have been proposed.

## Introduction

The hippocampus is a complex neural structure shown to be essential for memory across the lifespan. Animal and postmortem studies in humans reveal that the hippocampus shows significant development during the postnatal period, including cell formation (neurogenesis), cell death, cell migration, and synapse formation (Serres, 2001; Lavenex and Banta Lavenex, 2013). In addition, connectivity between cells within the hippocampus (e.g., the trisynaptic circuit, which connects functionally distinct subfields of the hippocampus) is not thought to reach maturity until the end of early childhood (approximately 5–7 years of age in humans; Serres, 2001; Lavenex and Banta Lavenex, 2013). Collectively these developmental changes in the hippocampus have been theoretically associated with the emergence of more robust memory ability (Nadel and Zola-Morgan, 1984; Pillemer and White, 1989; Lavenex and Banta Lavenex, 2013; Bauer, 2014). However, clear evidence supporting this in humans is only just emerging.

At present, studies in children examining the relation between hippocampal development and memory have primarily focused on structural development. Specifically, volume of hippocampus has been examined along the longitudinal axis (i.e., subregions) and related to variations in age and memory performance (e.g., DeMaster et al., 2013; Riggins et al., 2015, 2018; Schlichting et al., 2016; Daugherty et al., 2017). Overall, these studies show that hippocampal subregion volume varies as a function of age. However, findings regarding associations between hippocampal subregion volume and memory are inconsistent. First, in some studies, a significant correlation between hippocampal subregion volume and memory is reported in one age group (e.g., 4-year-old children), but not in other age groups (e.g., 6-year-old; Riggins et al., 2015). In other studies, one age group (e.g., 8–11-year-old) may show an association in one subregion of the hippocampus (e.g., tail), whereas another age group (e.g., adults) may show relations between volume and memory in different subregions (e.g., head and body; DeMaster et al., 2013). Still, other studies have shown that the direction of the correlation between volume and memory varies between ages, with younger participants showing a negative relation and with older participants showing a positive relation (Schlichting et al., 2016). Finally, some studies fail to find associations with hippocampal subregions at all (e.g., 8–25-year-old, Daugherty et al., 2017; 4–8-year-old, Riggins et al., 2018). These discrepancies may come from a variety of sources ranging from differences in the ranges of ages examined, differences in the use of various memory strategies or component processes, or differences in secondary cognitive resources and brain structures, as episodic memory is not an isolated process. However, an additional possibility is that the measure used (i.e., size/volume of subregions) may not directly relate to function.

Although volumetric measures are sensitive to overall changes in the size of the developing hippocampus, they do not provide any information about the underlying neurophysiological changes that might occur in hippocampal tissue, which may account for some of the variations in findings observed across studies. In contrast, diffusion imaging provides information about the composition and density of the brain tissue versus only its size (Le Bihan, 2003, 2014; Hansen et al., 2013). Generally, diffusion imaging is sensitive to the diffusion of water molecules, which reflects the underlying microstructural architecture of neural and glial processes and affords the ability to address questions relating to synaptic, dendritic, axonal, and glial densities and connectivity within brain tissue (Le Bihan, 2003, 2014; Hansen et al., 2013). Although the majority of diffusion imaging studies have focused on white matter, a growing body of research has focused on quantifying diffusion in cortical and subcortical gray matter regions (Salminen et al., 2016; Mah et al., 2017; Assaf, 2018). While multiple tensor-based measures are often computed for white matter (i.e., FA, RD, and AD), in generally isotropic and less directionally cohesive tissue like gray matter, mean diffusivity (MD) is the preferred diffusivity measure. Gray matter diffusivity is generally less dependent on the orientation of the underlying tissue, and thus MD is a more reliable and interpretable measure for gray matter tissue integrity (Basser and Jones, 2002).

Relevant to the present study, hippocampal MD is associated with alterations in synaptic, glial, and dendritic densities, such as swelling, increased arborization, or synaptic pruning within the hippocampus (Blumenfeld-Katzir et al., 2011; Sagi et al., 2012; Crombe et al., 2018; Stolp et al., 2018). For example, in older populations, age is associated with increased hippocampal MD, which is thought to result from a decline in dendritic and synaptic densities, as well as a general decline in hippocampal gliogenesis, neurogenesis, synaptogenesis, and angiogenesis (den Heijer et al., 2012; Pereira et al., 2014; Wolf et al., 2015; O’Shea et al., 2016). In contrast, development shows a different trend, with several studies reporting that hippocampal MD decreases with age during infancy, childhood, and adolescence, which are believed to be driven by increases in neurite density (i.e., higher axonal density, neurite density, and increased myelination), as opposed to increased axonal coherence (Forkert et al., 2016; Mah et al., 2017; Fjell et al., 2019). To combine the aging and developmental literatures, Langnes et al. (2019a) examined variations in microstructure of the hippocampus across the lifespan (4–93 years). They reported distinct trajectories for anterior versus posterior hippocampal subregions. Specifically, the anterior hippocampus showed a protracted period of development continuing well-into adulthood, whereas posterior development was completed in early childhood. Moreover, age-related differences were more evident in microstructure than they were in volume, suggesting that MD may be a more sensitive measure of developmental changes in hippocampal integrity.

Thus, developmental differences in the composition of brain tissue as measured through diffusion imaging may be more directly related to neurophysiological and microstructural changes that are believed to play a role in producing a normal hippocampal-dependent memory function (Bourne and Harris, 2008; Blumenfeld-Katzir et al., 2011; Sagi et al., 2012; Leal and Yassa, 2015). Evidence supporting this possibility comes from both aging and developmental groups. First, in adults, hippocampal MD is a stronger and earlier predictor of hippocampal-dependent memory decline than hippocampal volume (Kantarci et al., 2005; Fellgiebel et al., 2006; Fellgiebel and Yakushev, 2011; van Norden et al., 2012; Aribisala et al., 2014; Wolf et al., 2015). In a longitudinal study, Kantarci et al. (2005) found that hippocampal diffusion, but not hippocampal volume improved the accuracy when predicting conversion of individuals with mild cognitive impairment to Alzheimer’s disease. More recently, Aribisala et al. (2014) found in over 500 older adults that hippocampal MD was a significant predictor of memory, cognitive processing speed, and fluid intelligence, whereas only left hippocampal volume predicted memory performance (Kantarci et al., 2005; Aribisala et al., 2014). In development earlier in life, MD in anterior hippocampus has been related to verbal memory performance across both shorter (30-min, 4–93-year-old; Langnes et al., 2019a) and longer (average 9 days, 4–25-year-old; Fjell et al., 2019) delays, suggesting that diffusion measures can be associated with function (i.e., behavior).

Results showing associations between hippocampal diffusion and memory are exciting as they suggest nuanced measures of hippocampal integrity, which take differences in underlying tissue composition, as opposed to size into account, may be particularly informative across development. However, more work is needed to better understand relations between hippocampal microstructure and memory, particularly during early childhood when changes in hippocampal circuitry are thought to be most dramatic. As described above, development of connectivity within the hippocampus is thought to continue until at least the end of early childhood (i.e., 5–7 years of age in humans, Serres, 2001; Lavenex and Banta Lavenex, 2013), and has been argued to coincide with the emergence of more robust memory.

The purpose of this study was 2-fold: first, to examine age-related differences in hippocampal microstructure (measured *via* diffusion) with a focus on early childhood; and second, to examine whether hippocampal microstructure relates to memory performance during this developmental period. Memory for details (i.e., the source from whom children learned novel facts) was elected as the variable of interest, as it is thought to rely on children’s ability to bind details of previous experiences (Olsen et al., 2012; Yonelinas, 2013; Ekstrom and Yonelinas, 2020). Moreover, performance on such a task has been previously related to hippocampal subregion volume during this developmental period with mixed results (cf. Riggins et al., 2015, 2018). Because macrostructure (i.e., volume) of hippocampal subregions has been considered previously in the larger sample from which participants in the current report were included (Riggins et al., 2018), the present report focused on microstructure but included an analysis of hippocampal volume as a predictor and as a covariate. However, this analysis differs in its examination of anterior and posterior subregions versus delineating the hippocampus along its longitudinal axis into the subregions of head, body, and tail and focuses on the influence of hippocampal microstructure independent of previously used volumetric measures. Based on previous reports, we hypothesized that there would be a negative relation between hippocampal MD and age across early childhood and that lower hippocampal diffusion would predict better source memory performance, even after controlling for hippocampal volume.

## Materials and Methods

### Participants

The current study was a part of a larger research project examining the development of the brain in relation to episodic memory during early‐ to mid-childhood (see Riggins et al., 2018). This report examines a novel question regarding age-related differences in the microstructural integrity of the hippocampus with respect to source memory.

The study included 93 4–8-year-old children [*M* (SD) = 6.78 (1.35) years, reported males = 50]. The final sample of participants was predominantly Caucasian (59%), from middle‐ to high-income households (median => $105,000). Prior to data collection, all methods were approved by the Institutional Review Board at The University of Maryland. Children were screened to ensure that they were not born premature, had normal or corrected-to-normal vision, and had no diagnosis for any neurological conditions, developmental delays, or disabilities. Parents provided informed consent, and written assent was obtained for children older than 7 years of age.

### Behavioral Measures of Memory

Participants completed an established source memory task to examine relations between hippocampal volume and episodic memory (see Riggins et al., 2018, for additional details). This task is sensitive to age-related differences in memory ability during early childhood (Drummey and Newcombe, 2002; Riggins, 2014). Briefly, children visited the lab on two separate occasions. During the first visit, children watched digital videos in which they were taught 12 novel facts, six each from one of two different sources: a person or a puppet. Children were instructed to remember the facts but were not told they needed to pay attention to the source. During the second visit, approximately 1 week later [*M* (SD) = 7.35 (1.81) days], children were tested on their memory for both the novel facts and their source. Children were asked to answer 22 fact questions and to tell the experimenter where or from whom they had learned the answers to those questions. Six of the 22 facts had been presented by the person, six facts from the puppet, five were facts commonly known by children, and five were facts that children typically would not know.

After the experimenter asked each question, children were given the opportunity to answer freely. If children indicated they did not know the answer, they were given four pre-determined multiple-choice options. Once children gave an answer to the trivia question, they were asked where or from whom they had learned the information. As with fact questions, children were given the opportunity to answer freely, but if they indicated they did not know, where the fact was learned, given multiple-choice options: parent, teacher, person in the video, puppet in the video, or just knew/guessed.

The main dependent measure of interest for this report was source memory, which refers to the proportion of questions for which the child accurately recalled *both* the fact and the source of the fact as it is thought to require binding of the fact and source, which is an important aspect of episodic memory (Miller et al., 2013; Ritchey and Cooper, 2020). Consistent with previous research, memory for individual facts was also examined as were the errors children made regarding source judgments. Three types of errors occurred: children indicating they guessed or always knew the fact (termed guessed/knew errors), children indicating a person outside the experiment taught them (termed extra-experimental errors, e.g., teacher, parent, TV, and book), or children indicating the wrong experimental source taught them the fact (termed intra-experimental errors). These variables vary in their hypothesized relation to the hippocampus, and thus are not the focus of the present paper. However, we did examine relations between these memory measures and hippocampal MD. No significant relations were emerged.

### Brain Image Acquisition

Neuroimaging data were collected during children’s second visit to the lab. Children first took part in a mock scan that enabled them to get comfortable with the scanning environment. Children received motion training in the mock scan, were they practiced laying still and were given motion feedback by the experimenter. Following the mock scan, children completed the actual scan. During the scan, padding was placed around children’s heads to reduce motion. Participants were scanned in a Siemens 3.0 T scanner (MAGNETOM Trio Tim System, Siemens Medical Solutions, Erlangen, Germany) with a 32-channel coil. During scans, children watched a movie of their choosing to promote compliance.

Image acquisition included a high-resolution T1 magnetization-prepared rapid gradient-echo (MPRAGE) sequence (176 contiguous sagittal slices, 9 mm isotropic voxel size; 1,900 ms TR; 2.32 ms TE; 900 ms inversion time; 9-degree flip angle; 256 × 256 pixel matrix). T1 images were checked immediately following the scan to ensure high data quality. If the quality of the image was deemed to be too low, due to visual banding or visible blurring, the scan was repeated during the same session (*n* = 8).

Diffusion images were acquired with a twice-refocused spin-echo single-shot Echo Planar Imaging sequence with a parallel imaging mode (GRAPPA) at acceleration factor of 2. The diffusion scheme comprised of 64 non-collinear diffusion-weighted acquisitions with a value of *b* = 1,000 s/mm^2^ and a single T2-weighted *b* = 0 s/mm^2^ acquisition (TR/TE = 5,500/85 ms, 96 × 96 matrix, 2.2 × 2.2 mm^2^ in-plane resolution, flip angle = 90°, and a bandwidth of 1,158 Hz/Px, for all 44 slices at 3.5-mm thickness).

### Brain Image Processing

#### Magnetic Resonance Imaging

Processing of T1-weighted anatomical images was implemented with the FreeSurfer image analysis suite (http://surfer.nmr.mgh.harvard.edu/, version 6.0). The cross-sectional “recon-all” processing stream was implemented to perform initial motion correction, intensity normalization, and computation of the transformation to Talairach standard space, followed by non-brain tissue removal, cortical reconstruction, and volumetric segmentation of cortical and subcortical structures. FreeSurfer is a standard automatic segmentation program that is appropriate for use in children as young as 4 years of age (Ghosh et al., 2010). Automated hippocampal segmentation was performed for each participant’s T1-weighted image using the FreeSurfer hippocampal subregion segmentation program (Iglesias et al., 2015). This method uses a probabilistic atlas of the hippocampus based on ultra-high resolution, *ex vivo* MRI to produce automated segmentation of hippocampal subregions. An anterior hippocampal subregion was created from the automated hippocampal head segmentation, while the posterior hippocampal subregions were created by combining the automated hippocampal body and tail segmentations. This segmentation method for anterior and posterior hippocampi was chosen to preserve the spatial resolution of our diffusion imaging technique. This FreeSurfer segmentation algorithm separates the head and body *via* the uncus (the medial most region of the hippocampus; Iglesias et al., 2015). All automatic hippocampal segmentations were manually checked for accuracy, and one subject was removed from analysis due to poor hippocampal segmentation (female, 6.81 years).

#### Diffusion Tensor Imaging

Diffusion-weighted images were processed using tools in the FMRIB Software Library (FSL v6.0.2; Image Analysis Group, FMRIB, Oxford, United Kingdom; https://www.fmrib.ox.ac.uk/fsl/; Smith et al., 2004). Using the FMRIB Diffusion Toolbox, subject motion and eddy current-induced distortions were corrected (Andersson et al., 2003; Andersson and Sotiropoulos, 2016). Additionally, the average percentage of slices with suspect signal drop out due to head motion was less than 0.2% of all slices and was calculated using previously reported methods (Yendiki et al., 2014). A binary brain mask was created by removing the non-brain tissue with FSL’s Brain Extraction Tool (BET) from each subject’s non-diffusion (b0) weighted image volume. To correct for potential b0 inhomogeneities in the diffusion data and improve diffusion to anatomical co-registration, the Advanced Normalization Tool (ANT; Avants et al., 2008) was used. Specifically, the b0 image was registered to the bias-corrected, and skull stripped image using the antsIntermodalityIntrasubject.sh script, which uses ANT’s robust SyN nonlinear registration algorithm with a mutual information criterion that is optimized for within-subject registration across image modalities. This method has been shown to have equal, if not superior, performance to standard field map methods for correcting b0 inhomogeneities and aligning b0 and T1 images (Wang et al., 2017). The computed rigid and nonlinear registrations were then applied to the diffusion image. Next, eigenvector and eigenvalues along with MD were computed in native anatomical space using the dtifit program (Pierpaoli et al., 1996). All registrations between b0 and T1-weighted images were visually inspected, and no manual interventions were needed.

### Statistical Analyses

All analyses were run in R^1^ using Rstudio^2^ integrated development environment. Multiple linear regression was used for all analyses. First, to examine relations between age and hippocampal diffusivity, bilateral hippocampal MD was predicted *via* age and sex. Second, to verify previous relations between source memory performance and age (Riggins, 2014; Riggins et al., 2015, 2018), source memory performance was also predicted *via* age. Third, to examine relations between hippocampal MD and source memory, source memory performance was predicted *via* hippocampal MD, controlling for hippocampal volume. For comparison, the same analysis was conducted again to examine relations between hippocampal volume and source memory; in this case, source memory performance was predicted *via* hippocampal volume, controlling for hippocampal MD. Finally, to explore whether hippocampal MD was a significant predictor of source memory performance when controlling for age, sex, and hippocampal volume, a final analysis predicting source memory controlling for these variables was performed. All analyses were first conducted for whole bilateral hippocampus and, if significant, were followed-up by separate analyses for anterior and posterior subregions to determine presence of any regional specificity. As a check for outliers, all data points for all multiple linear regression models were tested for abnormal influence (Cook’s *D* > 0.5), leverage (hat value > 3 times average), and discrepancy (studentized residuals > 3). Based on an exclusionary criterion of violating more than one of these three heuristics, no data points had to be removed from any of the analyses. Additionally, predictors in all models had variance inflation factors less than 1.5 and collinearity tolerance values greater than 0.7, which indicates that there were no issues of multicollinearity.

## Results

### Age‐ and Sex-Related Differences in Hippocampal Diffusivity

**Figure 1:**
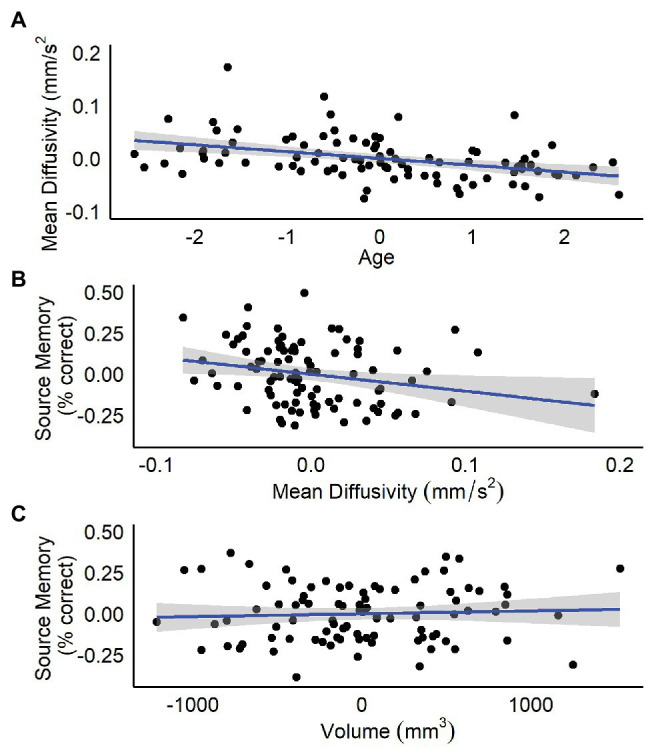
Residualized plots showing relations between **(A)** total bilateral hippocampal MD and age controlling for sex and total bilateral hippocampal volume, **(B)** total bilateral hippocampal MD and source memory controlling for total bilateral hippocampal volume, and **(C)** total bilateral hippocampal volume and source memory controlling for total bilateral hippocampal MD.

Results are summarized in Table 1. Age, but not sex, nor hippocampal volume was significantly related to bilateral hippocampal MD. Specifically, increased age was associated with lower MD (Figure 1A). Follow-up analyses by subregion revealed that, for the anterior hippocampus, age and anterior hippocampal volume were significant predictors (Table 1). However, for the posterior hippocampus, only age was a significant predictor (Table 1).

**Table 1:** Results of multiple linear regression predicting bilateral total, anterior, and posterior hippocampal mean diffusivity (MD) *via* age and sex, controlling for respective hippocampal volumes.

	Total MD	Anterior MD	Posterior MD
Predictor variables	*β*	*β*	*β*
Age	−0.41^***^	−0.27^**^	−0.34^**^
Sex	0.19^~^	0.09	0.07
Hippocampal volume	−0.04	−0.24^*^	−0.11
Adj. *R*^2^	0.20^***^	0.14^***^	0.13^**^
*F*	8.53^***^	6.01^***^	5.49^**^

Standardized beta coefficients are reported; ns, not significant; MD, mean diffusivity.

~ *p* < 0.10;

* *p* < 0.05;

** *p* < 0.01;

*** *p* < 0.001.

### Age‐ and Sex-Related Differences in Source Memory

Consistent with previous reports (e.g., Riggins, 2014; Riggins et al., 2015, 2018), age, but not sex, was a significant positive predictor of source memory (*β*_age_ = 0.42, *p* < 0.001 and *β*_sex_ = 0.01, *p* = 0.89). Specifically, older children performed better^3^.

### Age‐ and Sex-Related Differences in Hippocampal Volume

Age, but not sex, was a significant positive predictor of whole hippocampal volume [adjusted *R*^2^ = 0.10, *F*(2,89) = 6.15, *p* < 0.003, *β*_age_ = 0.31, *p* = 0.003, and *β*_sex_ = 0.19, *p* = 0.06; cf. Riggins et al., 2018]. Suggesting older children had larger whole hippocampal volumes. Additionally, follow-up analyses by subregion indicated a similar trend for anterior [adjusted *R*^2^ = 0.08, *F*(2,89) = 4.78, *p* = 0.011, *β*_age_ = 0.27, *p* = 0.008, and *β*_sex_ = 0.17, *p* = 0.09] and posterior hippocampal volumes [adjusted *R*^2^ = 0.09, *F*(2,89) = 5.47, *p* = 0.006, *β*_age_ = 0.30, *p* = 0.004, and *β*_sex_ = 0.18, *p* = 0.08], with older children having larger anterior and posterior hippocampal volumes.

### Relations Between Hippocampal Diffusivity, Source Memory, and Hippocampal Volume

Results are summarized in Tables 2 and 3. Total bilateral hippocampal MD was significantly related to source memory performance, even when controlling for hippocampal volume. Better performance on the source memory task was associated with lower MD (Figure 1B; Table 2). This relation was observed in both anterior and posterior hippocampal subregions; however, the former failed to reach conventional thresholds for significance (*p* = 0.066; Table 2). In contrast, hippocampal volumes were not found to be significant predictors of source memory when controlling for MD (total, anterior, and posterior hippocampi *ps* = 0.16, 0.59, and 0.11, respectively; Figure 1C). When all variables (age, sex, hippocampal MD, and hippocampal volume) were used to predict source memory performance, only age emerged as a significant predictor (Table 3). This was true in both anterior and posterior subregions.

**Table 2:** Results of multiple linear regression predicting source memory *via* total, anterior or posterior hippocampal MD controlling for respective hippocampal volumes.

	Source memory		
	Total	Anterior	Posterior
Predictor variables	*β*	*β*	*β*
Hippocampal volume	0.14	0.06	0.17
Hippocampal MD	−0.24^*^	−0.20^~^	−0.25^*^
Adj. *R*^2^	0.07^*^	0.03^~^	0.08^**^
*F*	4.20^*^	2.40^~^	5.15^**^

Standardized beta coefficients are reported; ns, not significant; MD, mean diffusivity.

~ *p* < 0.10;

* *p* < 0.05;

** *p* < 0.01.

**Table 3:** Results of multiple linear regression predicting source memory *via* total, anterior, or posterior hippocampal MD controlling for age, sex, and respective hippocampal volumes.

	Source memory		
	Total	Anterior	Posterior
Predictor variables	*β*	*β*	*β*
Age	0.36^**^	0.39^***^	0.35^**^
Sex	0.02	0.02	0.004
Hippocampal volume	0.05	−0.01	0.09
Hippocampal MD	−0.09	−0.09	−0.13
Adj. *R*^2^	0.15^**^	0.15^**^	0.16^**^
*F*	4.97^**^	4.87^**^	5.45^**^

Standardized beta coefficients are reported; ns, not significant; MD, mean diffusivity.

** *p* < 0.01;

*** *p* < 0.001.

## Discussion

The present study revealed that hippocampal MD was related to both age and memory performance in young children. Negative relations between age and hippocampal MD are consistent with previous developmental studies (e.g., Forkert et al., 2016; Mah et al., 2017; Fjell et al., 2019; Langnes et al., 2019a). Negative associations between hippocampal MD and source memory are novel and extend previous research examining hippocampal microstructure and verbal memory (Fjell et al., 2019; Langnes et al., 2019a) by documenting relations with source memory across a 1-week delay during the period of early to mid-childhood. Moreover, the present findings also suggest regional specificity in relations between MD and age, as associations appear stronger in posterior, compared to anterior subregions.

The appearance of a stronger relations between hippocampal MD and source memory in posterior hippocampus aligns with work showing stronger recruitment of the posterior subregion during the retrieval of associative memories in children compared to teenagers and adults (DeMaster et al., 2013; Langnes et al., 2019b). Functional specialization of anterior versus posterior hippocampus is still debated (see Poppenk et al., 2013, for review). However, these subregions are known to differ in terms of large-scale network connectivity with the rest of the brain, organization of entorhinal grid cells, and subfield compositions. Recent proposal has suggested that due to these differences, the anterior hippocampus is biased toward coarse, global representations, whereas the posterior hippocampus is biased toward fine-grained, local representations (Poppenk et al., 2013). Fine-grained details are precisely what are required for successful performance on the source memory task in this study, as children were required to recall which one of two similar sources had taught them a novel fact 1 week prior.

Finally, consistent with previous reports in adults, the present study suggests hippocampal MD may be a stronger predictor of hippocampal-dependent memory than hippocampal volume (Kantarci et al., 2005; Fellgiebel et al., 2006; Fellgiebel and Yakushev, 2011; van Norden et al., 2012; Aribisala et al., 2014; Wolf et al., 2015). This result is particularly exciting, as findings regarding relations between hippocampal volume and memory have been mixed in the developmental literature (DeMaster et al., 2013; Riggins et al., 2015, 2018; Schlichting et al., 2016; Daugherty et al., 2017). The present findings suggest hippocampal diffusion, which reflects differences in underlying tissue composition, as opposed to size (i.e., volume), may provide a precise and informative measure of functionally relevant development of the hippocampus during early childhood. This may be because diffusion imaging attempts to characterize the underlying physiological alterations in synaptic, glial, and dendritic densities (Blumenfeld-Katzir et al., 2011; Sagi et al., 2012; Mah et al., 2017; Crombe et al., 2018; Stolp et al., 2018) in a different manner than volume, by probing at the density and integrity of the hippocampal microarchitecture instead of gross changes in size or shape. For example, Mah et al. (2017) found that age was negatively associated with hippocampal and cortical MD in 8–13-years-old, which was highly associated (*r* > 0.8) with their measure of neurite density. This suggests that lower MD in development may be the result of changes in axonal density, dendritic density, and myelination within the hippocampus. These physiological alterations are thought to develop in early childhood and are hypothesized to be related to memory ability (Serres, 2001; Lavenex and Banta Lavenex, 2013); however, it is important to note that neither hippocampal volume nor MD can directly or independently measure any of these alterations.

Specifically, neuroanatomical work suggests that hippocampal development consists of increases in the number of cells, synapses, and connectivity (Serres, 2001; Lavenex and Banta Lavenex, 2013). These physiological changes would lead to a more dense tissue microstructure that would greater restrict the free movement of water molecules, and thus lower diffusion measures like MD. Differences in diffusivity may reflect not only variation due to typical changes as a function of maturation, but also differences between individuals of the same age, such that, those with a “tighter” structure show better memory performance, perhaps due to increased cell number or increased connectivity through higher synaptic or dendritic density.

It is notable that when both age and source memory were examined in relation to hippocampal MD, only age remained a significant predictor. This suggests maturation may have been underlying both reported effects. Because source memory improves with age, these measures are related and share variance. As a result, in the present report, we were unable to disentangle differences in memory due to age from differences in memory un-related to age (experience-related effects or individual differences). Future research examining one age group may be better suited to probe how individual differences in hippocampal MD relate to memory during this period.

The present study contributes important data regarding associations between the hippocampus and memory during early childhood. Strengths include the large sample size in a typically under-represented age group in neuroimaging studies, use of an established source memory task, and the inclusion of measures of both micro‐ and macro-structure. Despite these strengths, several limitations should be noted. First, findings are based on a cross-sectional sample, thus developmental changes cannot be concluded. Second, although diffusion measures provide insight into hippocampal microstructure that is inaccessible with volumetric measures, the spatial resolution of DTI in humans precludes the ability to infer individual physiological processes. Additionally, due to the nature of MD measuring average diffusivity in a voxel, it is not possible to parse out the individual contribution that different physiological processes and biological components have on the average diffusion signal. While MD has been shown to reflect neural plasticity and development, it is likely that differences in MD are related with a host of microstructural alterations that cannot be discerned with current human imaging techniques.

## Conclusion

The present study expands current understanding of relations between the hippocampus and memory in early childhood, a period characterized by dramatic changes in both. These data shed additional light on the role of the hippocampus, a complex neural structure, and how it relates to memory during childhood. Future work needs to explore the relationship between hippocampal diffusivity and additional measures of memory development across varying developmental periods. Additionally, future longitudinal studies are needed to discern a causal relationship between hippocampal diffusivity and memory development.

## Data Availability Statement

The data that support the findings of this study are available from the corresponding author upon reasonable request. Requests to access the datasets should be directed to riggins@umd.edu.

## Ethics Statement

The studies involving human participants were reviewed and approved by University of Maryland Internal Review Board. Written informed consent to participate in this study was provided by the participants’ legal guardian/next of kin.

## Author Contributions

TR and KC carried out the experiment. DC analyzed the data and wrote the manuscript with support from KC and TR. DC and TR conceived the original idea. TR supervised the project. All authors contributed to the article and approved the submitted version.

### Conflict of Interest

The authors declare that the research was conducted in the absence of any commercial or financial relationships that could be construed as a potential conflict of interest.

## Footnotes

^1^https://www.r-project.org

^2^www.rstudio.com

^3^Item memory was also assessed and is defined as memory for facts regardless of contextual information. Item memory was measured as the proportion of facts correctly remembered, irrespective of source memory. Consistent with prior reports (e.g., Riggins, 2014; Riggins et al., 2015, 2018), age, but not sex, was also a significant positive predictor of memory for facts regardless of contextual information (*β*_age_ = 0.51, *p* < 0.001 and *β*_sex_ = 0.00, *p* = 0.96). Additionally, a partial correlation analysis controlling for age suggests that source memory and item memory were significantly correlated (*r* = 0.60, *p* < 0.001).
